# Esophageal intramural pseudodiverticulosis characterized by barium esophagography: a case report

**DOI:** 10.1186/1752-1947-4-145

**Published:** 2010-05-21

**Authors:** Owen J O'Connor, Adrian Brady, Fergus Shanahan, Eamonn Quigley, Michael O'Riordain, Michael M Maher

**Affiliations:** 1 Department of Radiology, Mercy University Hospital, Cork, Ireland; 2 Department of Medicine, University College Cork, Cork, Ireland; 3 Department of Surgery, Mercy University Hospital, Cork, Ireland

## Abstract

**Introduction:**

Esophageal intramural pseudodiverticulosis is a rare condition characterized by the dilatation of the submucosal glands.

**Case presentation:**

We present a case of esophageal intramural pseudodiverticulosis in a 72-year-old Caucasian man who presented with dysphagia and with a background history of alcohol abuse. An upper gastrointestinal endoscopy of our patient showed an esophageal stricture with abnormal mucosal appearances, but no malignant cells were seen at biopsy. Appearances on a barium esophagram were pathognomonic for esophageal intramural pseudodiverticulosis.

**Conclusion:**

We demonstrate the enduring usefulness of barium esophagography in the characterization of abnormal mucosal appearances at endoscopy.

## Introduction

Esophageal intramural pseudodiverticulosis is a rare condition characterized by the dilatation of submucosal glands. Based on approximately 250 cases reported to date, this condition is slightly more common in men than in women [1,2]. Intramural pseudodiverticulosis is most commonly associated with gastrooesophageal reflux and esophagitis and less commonly with alcoholism, diabetes mellitus, Crohn's disease, tuberculosis, Mallory-Weiss syndrome and achalasia [3,4]. The average age at presentation is 54 years and patients typically present with dysphagia, which is frequently associated with stricture formation, as in case we describe here [3,4]. Symptoms usually respond well to anti-inflammatory medication and balloon dilatation of strictures.

## Case presentation

**Figure 1:**
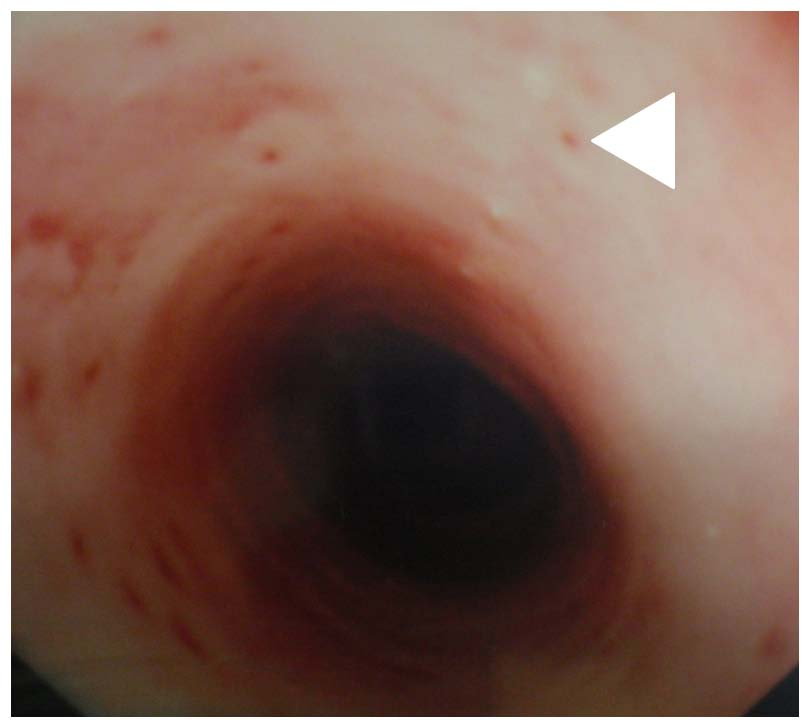
**Endoscopic image of the esophagus**. The pseudodiverticular orifices appear as multiple erythematous macules on the surface of the esophagus.

**Figure 2:**
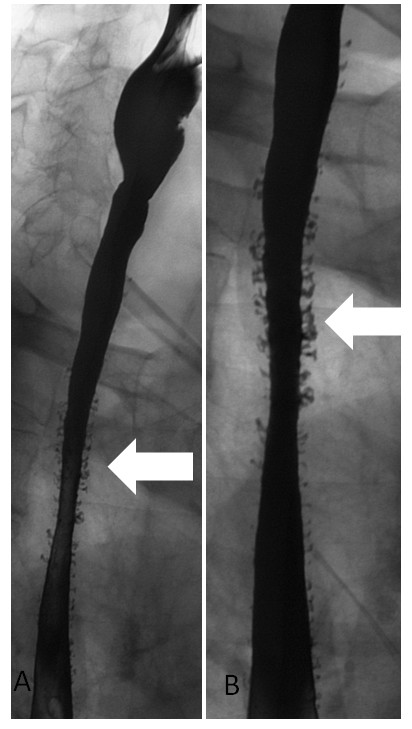
**Barium oesophagram**. **(A) **There are numerous barium-filled diverticula of the mid- and lower esphagus. **(B) **There is lack of distension of the mid-esophagus, thus indicating the presence of a stricture.

A 72-year-old Caucasian man (height: 170 cm, weight: 85 kg) presented with a 4-year history of mild dysphagia for solid foods. His medical history was notable for alcohol abuse and associated alcoholic hepatitis. An upper gastrointestinal endoscopy was initially performed on our patient. At endoscopy, a stricture of the mid-esophagus with numerous tiny erythematous macules on the mucosal surface was seen (Figure 1). There was clinical uncertainty about the cause of the stricture and the mucosal appearances. A barium esophagogram was performed (Figures 2A and 2B). Barium esophagogram demonstrated a smooth stricture of the mid-esophagus with numerous small (2 mm to 4 mm), flask-shaped outpouchings of the esophageal wall, an appearance which is pathognomonic for esophageal intramural pseudodiverticulosis [5].

## Discussion

Biphasic esophagography is generally recommended for the evaluation of an esophageal stricture. While a single contrast barium esophagogram provides maximal esophageal dilatation and is useful for the depiction of a stricture, a double contrast examination allows accurate imaging of the esophageal mucosa and wall. Barium esophagram is valuable for establishing this particular diagnosis as reports in the literature have suggested that the diverticular orifices are detected at endoscopy in only 20% of patients [2].

## Conclusion

This case demonstrates the enduring usefulness of barium esophagography in the characterization of peculiar mucosal appearances at endoscopy.

## Consent

Written informed consent was obtained from our patient for publication of this case report and any accompanying images. A copy of the written consent is available for review by the Editor-in-Chief of this journal.

## Competing interests

The authors declare that they have no competing interests.

## Authors' contributions

OJOC prepared the manuscript. MOR performed upper gastrointestinal endoscopy. AB and MM performed barium esophagography. FS and EQ medically managed our patient. All authors read and approved the final manuscript.
